# Supplementary material for: N-terminal tyrosine of ISCU2 triggers [2Fe-2S] cluster synthesis by ISCU2 dimerization
Source: Nat Commun. 2021 Nov 25;12:6902. doi: 10.1038/s41467-021-27122-w (PMC8617193; doi:10.1038/s41467-021-27122-w)
Supplement: Supplementary file 1 — Supplementary Information [file 41467_2021_27122_MOESM1_ESM.pdf]

Supplementary Information

N-terminal tyrosine of ISCU2 triggers [2Fe-2S] cluster synthesis by ISCU2 dimerization

Sven-A. Freibert[1,2,§], Michal T. Boniecki[3,§], Claudia Stümpfig[1], Vinzent Schulz[1], Nils Krapoth[1], Dennis R. Winge[1,4], Ulrich Mühlenhoff[1], Oliver Stehling[1,2], Miroslaw Cygler[3,*] and Roland Lill[1,2,5*]

[1] Institut für Zytobiologie im Zentrum SYNMIKRO, Philipps-Universität Marburg, Karl-von-Frisch-Str. 14, 35032 Marburg, Germany
[2] Core Facility 'Protein Biochemistry and Spectroscopy', Karl-von-Frisch-Str. 14, 35032 Marburg, Germany
[3] Department of Biochemistry, Microbiology & Immunology, University of Saskatchewan, 107 Wiggins Rd, Saskatoon, Saskatchewan S7N 5E5, Canada
[4] Department of Medicine, University of Utah Health Sciences Center, Salt Lake City, UT, USA
[5] LOEWE Zentrum für Synthetische Mikrobiologie SynMikro, Hans-Meerwein-Str., 35043 Marburg, Germany

[*] Corresponding authors:
Roland Lill
E-mail: lill@staff.uni-marburg.de, phone: +49-6421-286 6449

Miroslaw Cygler
E-mail: miroslaw.cygler@usask.ca, phone: +1-306-966-4361

[§] Sven-A. Freibert and Michal T. Boniecki contributed equally

Supplementary figure 1

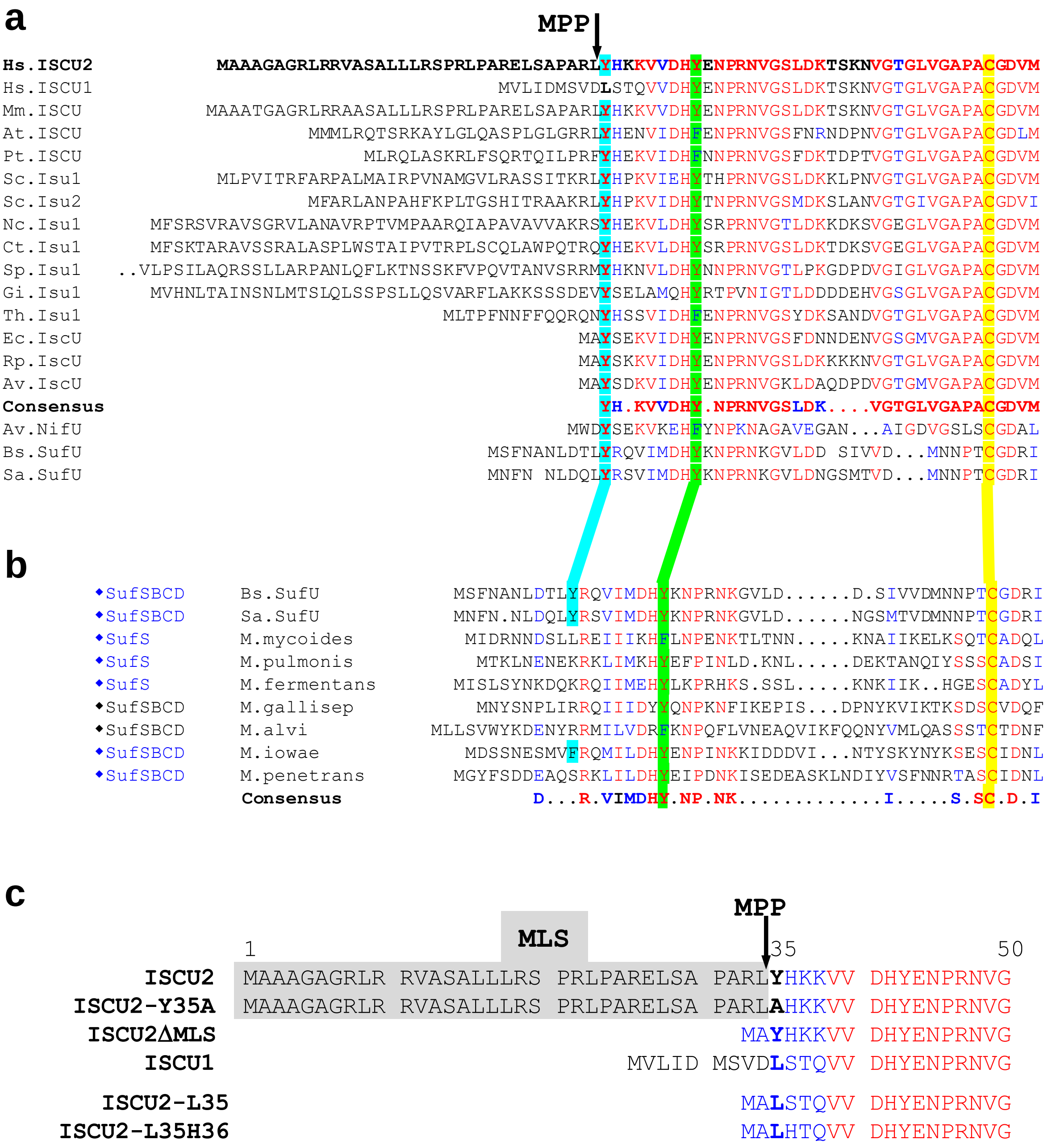

Supplementary Fig. 1: Amino acid sequence alignment of ISCU2 and related proteins. a: The multi-sequence alignment of the N-terminal region of human ISCU2 and related proteins including members of the bacterial NifU and SufU families was created with Multalin[1]. The cleavage site of mitochondrial matrix processing peptidase (MPP) in human ISCU2 is indicated by an arrow. All known mitochondrial and bacterial IscU-like scaffold proteins and the N-terminal domain of NifU-like proteins harbor a conserved N-terminal Tyr residue (cyan; Tyr35 in human ISCU2). In contrast, human cytosolic ISCU1 does not contain this residue due to a splicing event[2]. A conserved Tyr (green) and the first conserved Cys (yellow) are further highlighted. Abbreviations: Mitochondrial proteins: Hs, *Homo sapiens*; Mm, *Mus musculus*; At,

Arabidopsis thaliana; Pt, *Populus trichocarpa*; Sc, *Saccharomyces. cerevisiae*; Nc, *Neurospora crassa*; Ct, *Chaetomium thermophilum;* Sp, *Schizosaccharomyces pombe*. Mitosomal proteins[3]: Gi, *Giardia intestinalis*; Th, *Trachipleistophora hominis*. Bacterial proteins: Ec, *Escherichia coli* K12; Rp, *Rickettsia prowazekii*; Av, *Azotobacter vinelandii*; Bs, *Bacillus subtilis*; Sa, *Staphylococcus aureus*. **b:** The multi-sequence alignment of the N-terminal region of *Mycoplasma* SufU sulfur transferases was generated as in a, also showing the Bs.SufU and Sa.SufU sequences from part a for comparison. As outlined recently, most *Mycoplasmas* do not perform Fe/S protein biogenesis, and just contain SufS (no SufU) or SufS plus SufU (indicated on the left) to assist tRNA thiolation[3]. Only a few *Mycoplasma* strains of the Pneumonia subgroup additionally contain the SufBCD scaffold complex as indicated on the left. The N-terminal Tyr35 of human ISCU2 is not conserved in these *Mycoplasma* SufU proteins that do not perform a Fe/S scaffold function. **c:** The N-terminal regions of the human ISCU2-related proteins used for complementation in the *in vivo* studies of Fig. 1 and the structurally and biochemically studied mutant proteins ISCU2-L35 and ISCU2-L35H36 (for comparison the sequence of cytosolic ISCU1 is shown). The mitochondrial localization sequence (MLS) is highlighted in grey.

Supplementary figure 2

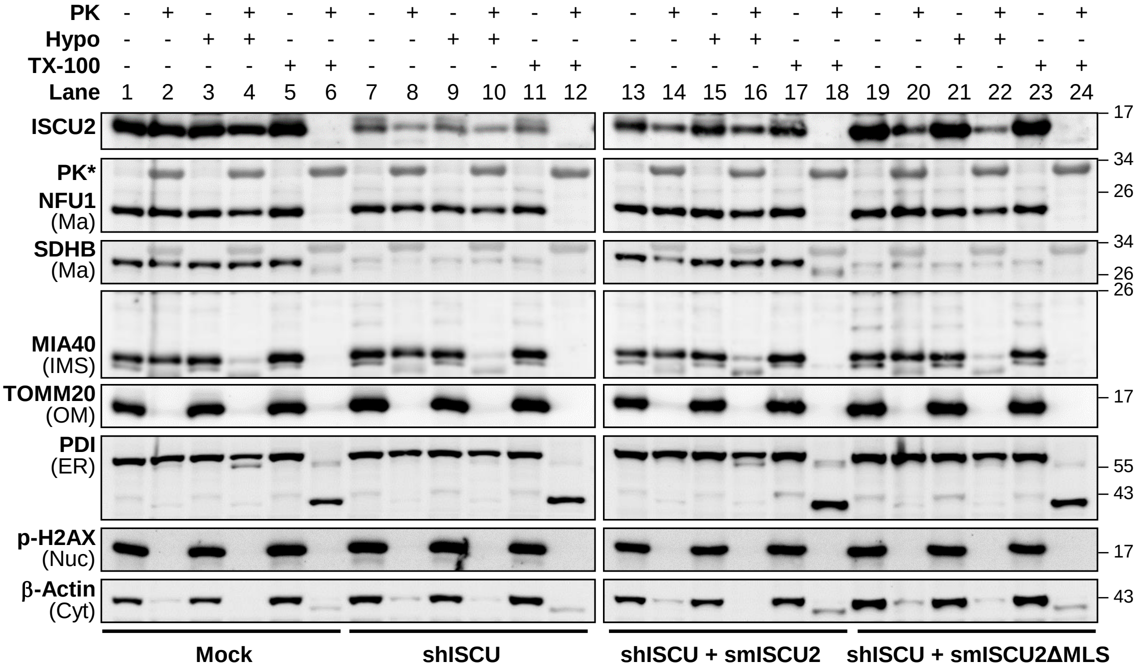

Supplementary Fig. 2: Sub-cellular localization of plasmid-expressed ISCU2 proteins. Mock- or RNAi-treated (shISCU) HeLa cells expressing either no protein (-), smISCU2 or smISCU2ΔMLS were cultivated for 3 days (cf. Fig. 1) followed by digitonin fractionation. Aliquots of the mitochondria-containing membrane fraction were additionally treated with hypotonic buffer (Hypo) to rupture the outer membrane (OM), or with the detergent Triton X-100 (TX-100) to completely lyse the mitochondria. Untreated and treated samples were subjected to proteinase K digestion (PK*; non-specifically stained by the anti-NFU1 antiserum) to determine the protease sensitivity of the analyzed proteins by immunostaining. Without treatment only cytosol-exposed OM proteins (TOMM20) are sensitive to PK. Hypotonic treatment additionally renders proteins of the intermembrane space (IMS) PK-sensitive (MIA40). All proteins including matrix (Ma) proteins (NFU1, SDHB) become PK-sensitive only after detergent lysis. Endogenous ISCU2 is predominantly located in the matrix, yet a fraction is sensitive to PK indicating its attachment to the outside of mitochondria, possibly due to inefficient import. This is particularly true for plasmid-expressed smISCU2ΔMLS indicating that the latter protein has not reached the matrix, a conclusion also evident from the functional analyses of Fe/S protein levels and enzyme activities (cf. Fig. 1b,c). Input controls are nuclea r (Nuc) phosphorylated gamma histone 2AX (p-H2AX) and endoplasmic reticulum (ER) protei n disulfide isomerase (PDI). A fraction of β-actin is membrane-associated and was used as in put control for cytosol (Cyt). Representative blots are shown (n=3 biological replicates).

Source Data are provided as a Source Data file.

Supplementary figure 3

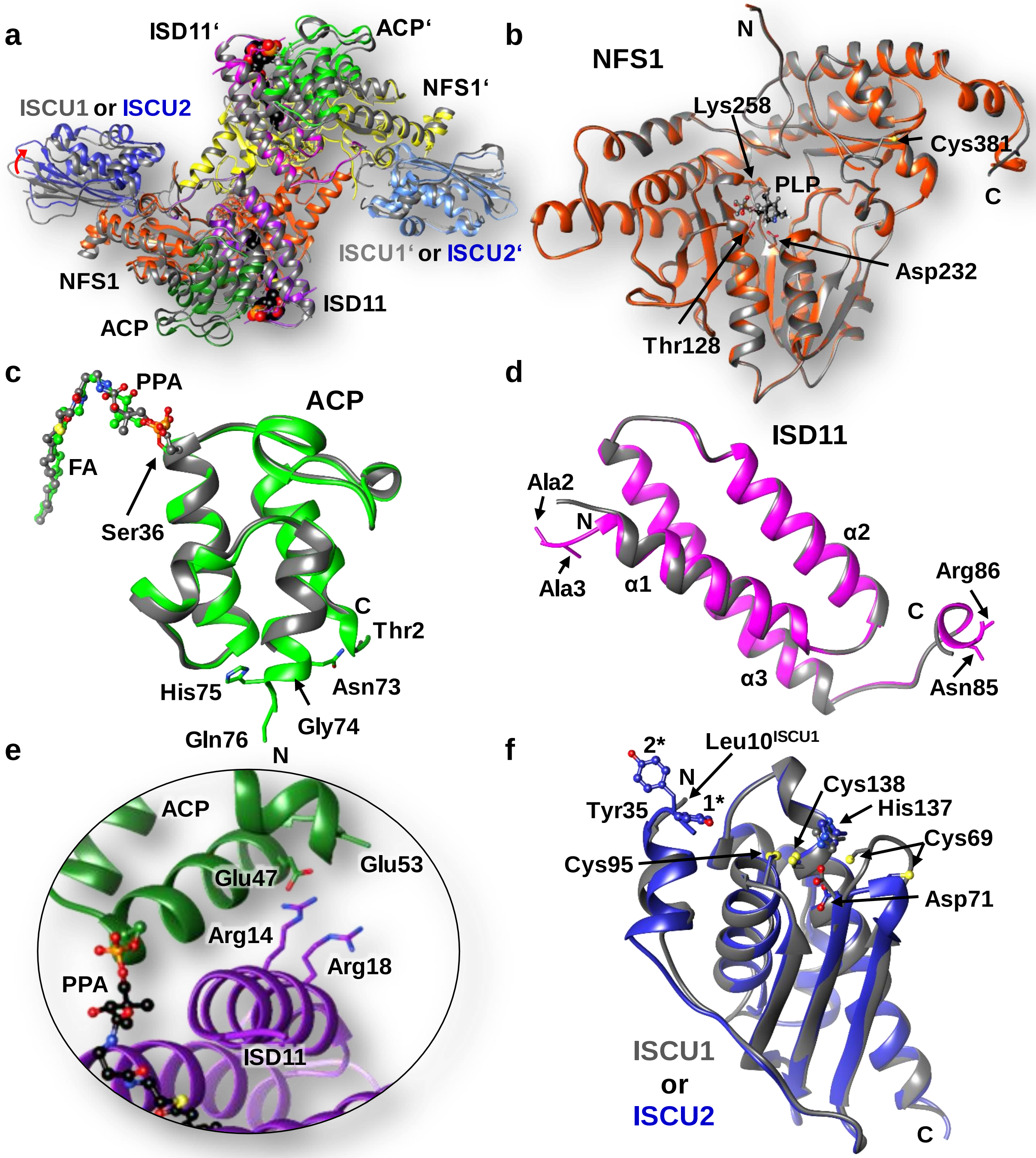

Supplementary Fig. 3: The high resolution structure of $(NIAU2)_2$ shows characteristic differences to that of $(NIAU1)_2$. a: Superposition of the $(NIAU2)_2$ and $(NIAU1)_2$ complexes. Color code for $(NIAU2)_2$ as in Fig. 2a. $(NIAU1)_2$ is shown in grey. For crystallographic details see Supplementary Tab. 2. **b:** Superposition of the NFS1 subunits of $(NIAU2)_2$ (orange) and $(NIAU1)_2$ (grey). **c:** Superposition of the ACP subunits of $(NIAU2)_2$ (green) and $(NIAU1)_2$ (grey). **d:** Superposition of the ISD11 subunits of $(NIAU2)_2$ (magenta) and $(NIAU1)_2$ (grey). **e:** Section of the ISD11-ACP sub-complex in $(NIAU2)_2$ with subunits colored as above. Residues Arg14

and Arg18 (depicted as sticks) of ISD11 form two ion pairs with Glu47 and Glu53 of ACP, respectively. These interactions are not resolved in the $(NIAU1)_2$ structure. **f:** Superposition of ISCU2 (blue) and ISCU1 (grey) of the respective $(NIAU)_2$ structures. Conserved Cys, His and Asp residues of the active center as well as the N-terminal $Tyr35^{ISCU2}$ and the corresponding $Leu10^{ISCU1}$ are depicted as sticks. The two conformers of $Tyr35^{ISCU2}$ are indicated as 1* and 2*.

Supplementary figure 4

a

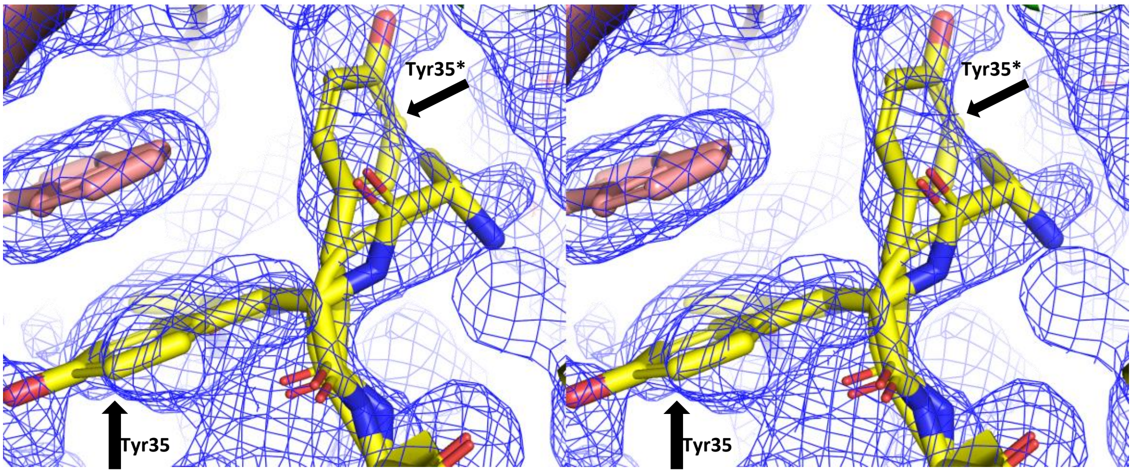

b

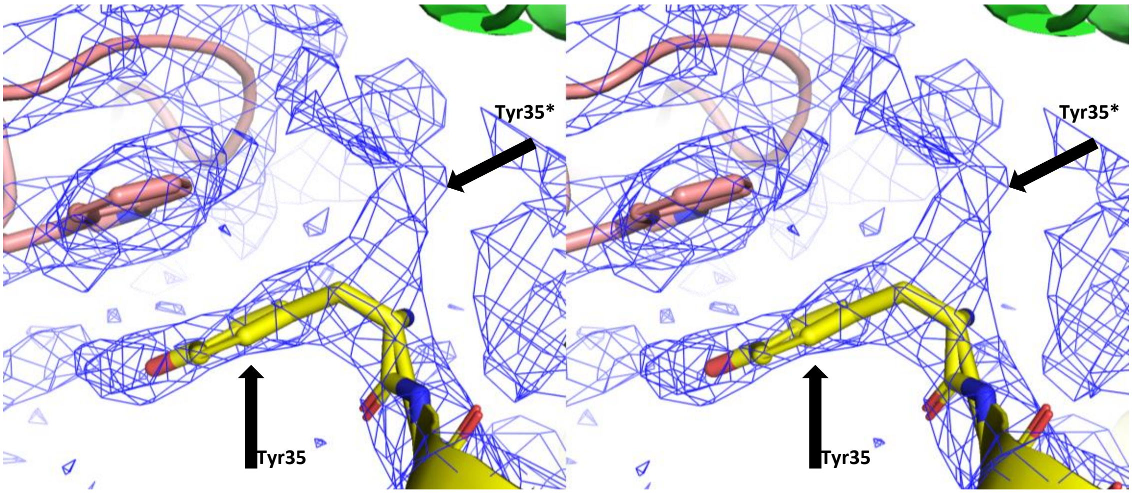

Supplementary Fig. 4: Stereo views to compare the flexible N-terminus of ISCU2 in X-ray and cryo-EM structures. a) Structure and map at 1.0 sigma from X-ray data at 1.57 Å resolution, PDB ID: 6UXE [https://www.rcsb.org/structure/6UXE]. **b)** The electron density 'omit' map 2Fo-DFc at 1.0 sigma from the cryo-EM structure at 3.2 Å resolution, PDB ID: 6NZU [https://www.rcsb.org/structure/6NZU]. Yellow, ISCU2, Magenta NFS1' with highlighted W97. The cryo-EM map shows the possible second conformation of the N-terminal $Y35^{ISCU2}$ residue (Tyr35*) in addition to the published one (Tyr35).

Supplementary figure 5

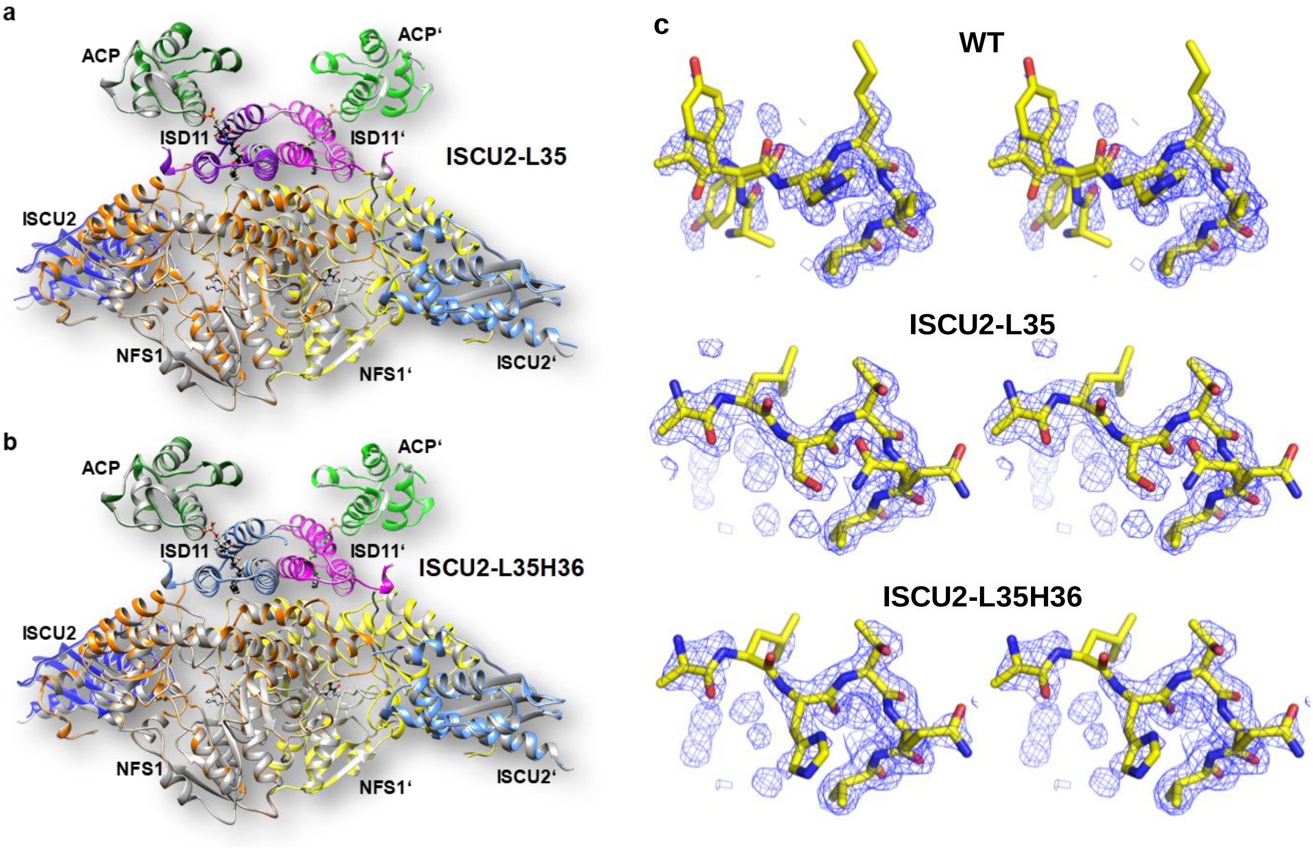

Supplementary Fig. 5: High resolution structures of $(NIAU2)_2$ complexes with N-terminally altered ISCU2 proteins. X-ray structures of **a)** $(NIAU2\text{-}L35)_2$ (1.95 Å resolution) and **b)** $(NIAU2\text{-}L35H36)_2$ (1.9 Å) are superimposed on the $(NIAU1)_2$ structure (grey). Color codes as in Fig. 2a. For crystallographic details see Supplementary Tab. 2. **c)** Stereo views for Fig. 2c. Top panel shows wild-type (WT) ISCU2 taken from $(NIAU2)_2$ (PDB ID: 6W1D [https://www.rcsb.org/structure/6W1D]). ISCU2-L35 (middle) and ISCU2-L35H36 (lower panel) are oriented in the same way as WT. The electron density 'omit' map 2Fo-DFc is displayed at 0.8 sigma level.

Supplementary figure 6

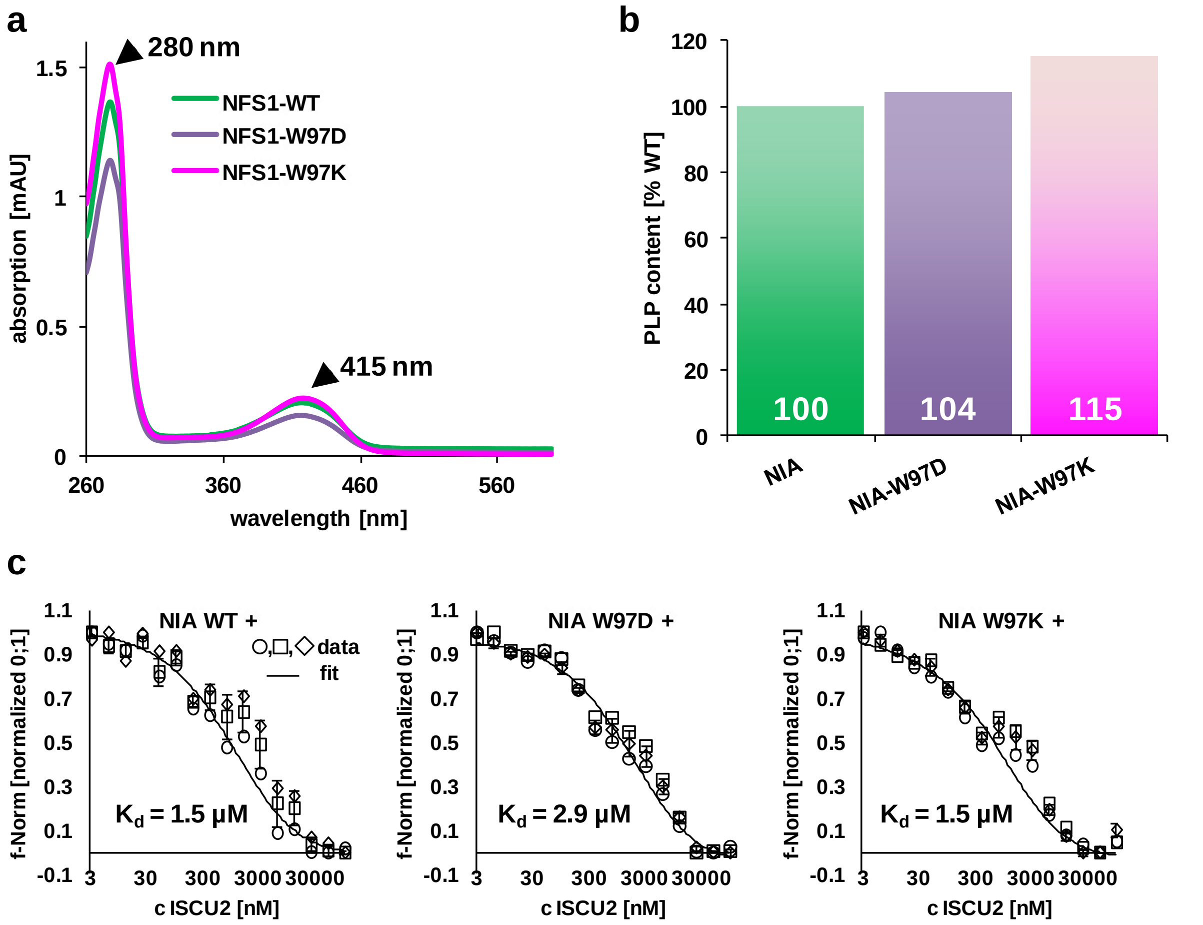

Supplementary Fig. 6: Conserved Trp97 of NFS1 does not contribute to the interaction with ISCU2. a,b: The pyridoxal-phosphate (PLP) content of purified human NFS1, NFS1-W97D, and NFS1-W97K, all as part of the $(NIA)_2$ complex was measured spectroscopically (**a**) and quantitated from the absorption at 415 nm normalized to the absorption at 280 nm taking into account the altered extinction coefficient of the Trp97 mutant proteins (**b**). **c:** Primary data for microscale thermophoresis measurements of Fig. 3a to determine the interaction between various $(NIA)_2$ complexes with ISCU2 as indicated. Solid line represents the fitted curve. For raw data see Source data file. (n=3 biological replicates, error bars show SD)

Supplementary figure 7

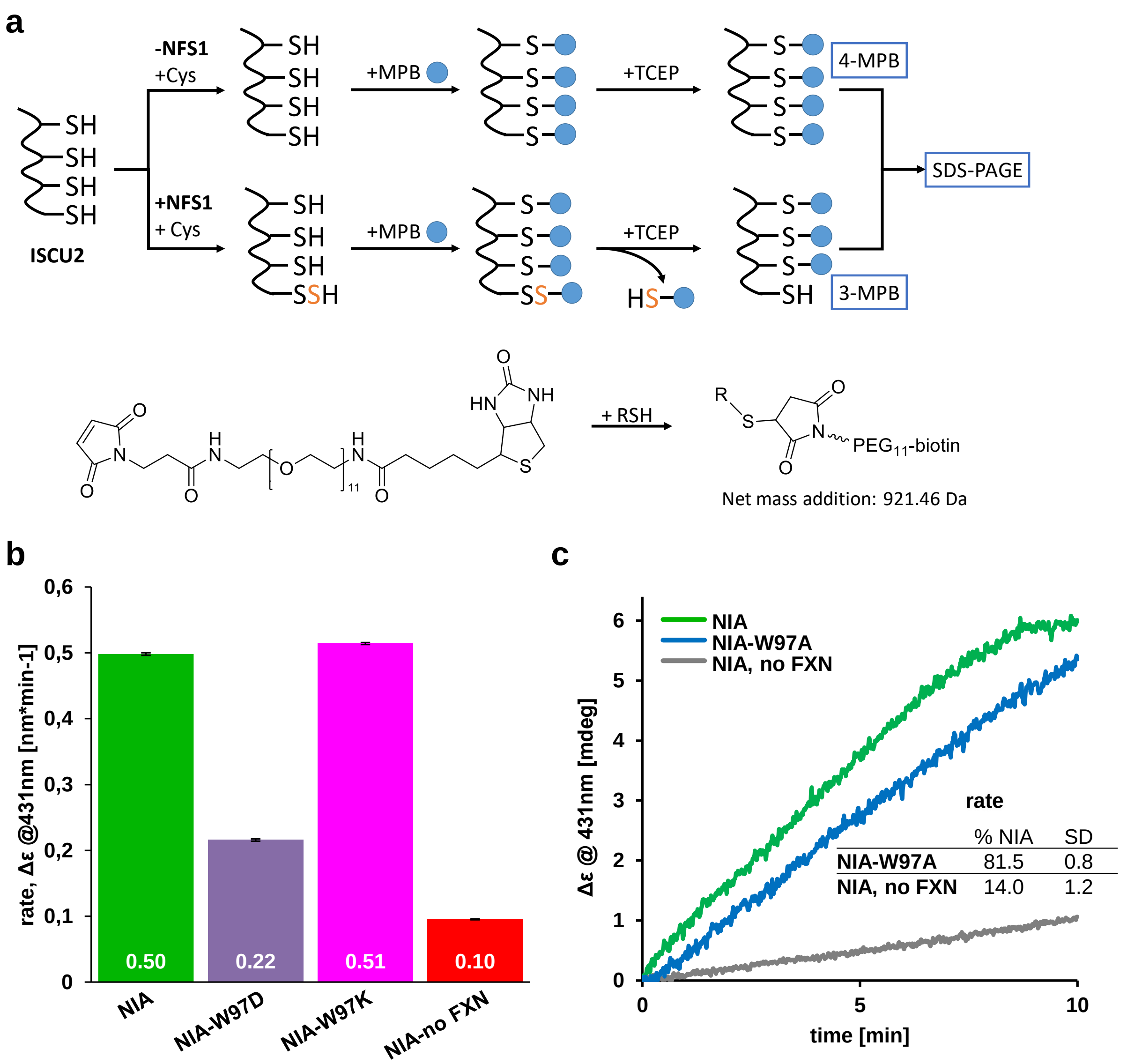

Supplementary Fig. 7: Conserved Trp97 of NFS1 does not play a critical role in Fe/S protein biogenesis. a: Cartoon for persulfide detection via an alkylation-based band-shift gel assay. Bottom: Maleimide-polyethylene-glycol$_{11}$-biotin (MPB) specifically reacts with free thiol groups including persulfides at neutral pH. Alkylation by MPB11 leads to a net mass addition of 921.46 Da per thiol. Top: ISCU2 contains four Cys residues one of which can be modified by persulfidation by NFS1 and free Cys. After labeling all Cys thiols and persulfides with MPB, ISCU2 is treated with TCEP for reduction of the persulfide. Samples are analyzed by SDS-PAGE to determine the mass shift for tetra- (4-MPB) or tri- (3-MPB)-modified thiols. **b:** Initial rates of enzymatic reconstitution of ISCU2 by NFS1 or the indicated NFS1-W97 mutant proteins (as part of the $(NIA)_2$ complex). Rates were calculated from the slopes between 1 and 6 min of representative data shown in Fig. 3d. Fitting was done using Origin 8G. The error bars represent SD of the fitting procedure. **c:** Enzymatic [2Fe-2S] cluster reconstitution (cf. Fig. 3d)

on ISCU2 with wild-type NFS1 (NIA) or the NFS1-W97A variant. In the negative control, FXN was omitted (NIA, no FXN). The rates (insert) are given relative to wild-type $(NIA)_2$, and were calculated as explained in part b.

Supplementary figure 8

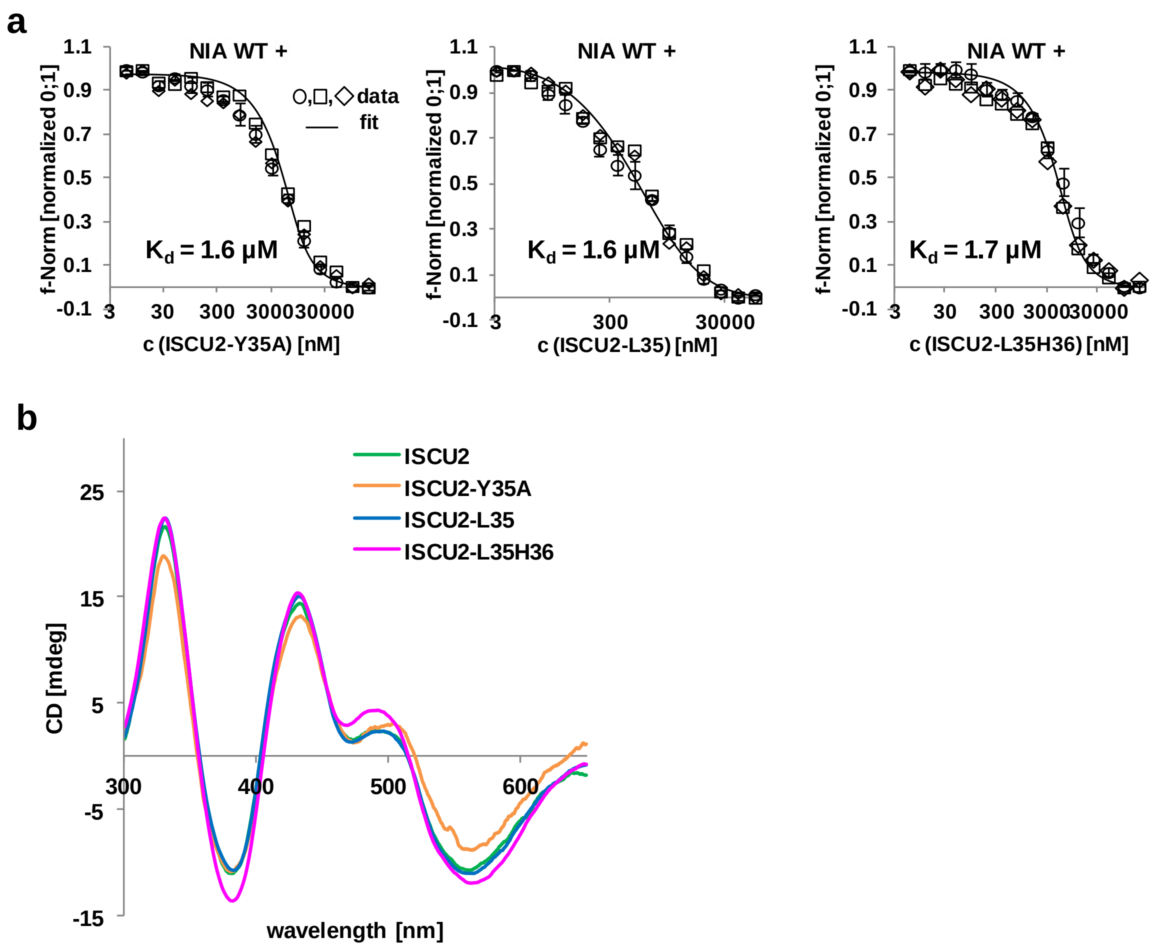

Supplementary Fig. 8: Tyr35 is not involved in cysteine desulfurase activation, persulfide reduction or cluster coordination. a: Primary data for microscale thermophoresis measurements of Fig. 4a to determine the interaction between $(NIA)_2$ complexes with various ISCU2 variants as indicated. Solid line represents the fitted curve. (n=3 biological replicates, error bars show SD) Data for wild-type ISCU2 see Supplementary Fig. 6c. For raw data see Source data file. **b:** CD spectra of chemically reconstituted ISCU2-Y35A, ISCU2-L35 and ISCU2-L35H36 (orange, blue and magenta, respectively) compared to ISCU2 (green). All variants can be chemically reconstituted and show CD spectra comparable to that of wild-type ISCU2. Spectra were normalized to protein concentration.

Supplementary figure 9

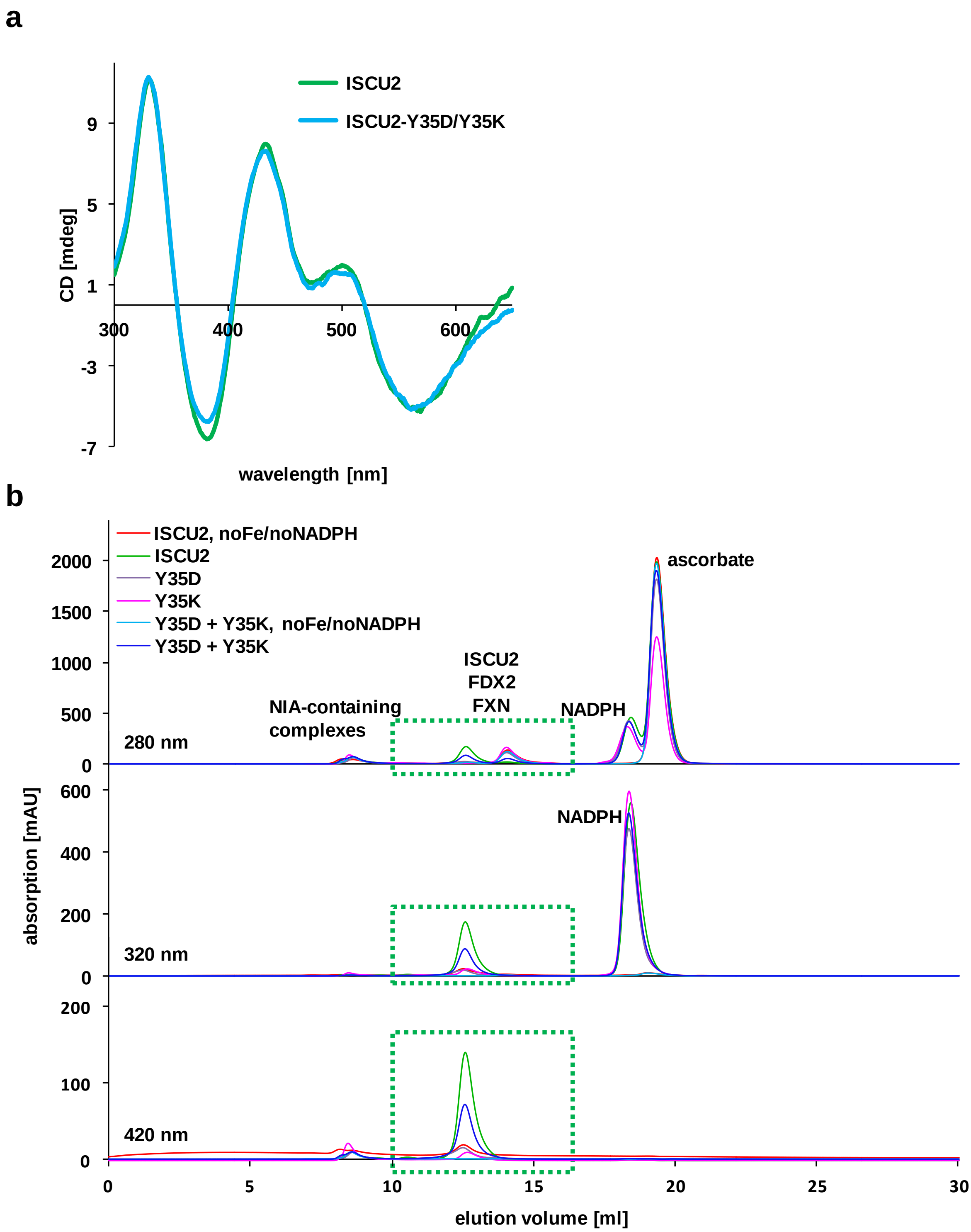

Supplementary Fig. 9: Dimerization of two ISCU2 requires their binding to different $(NIAU2)_2$ complexes. a: Wild-type ISCU2 or a mixture of ISCU2-Y35D and ISCU2-Y35K was enzymatically reconstituted for 20 min (cf. Fig. 5c). CD spectra were recorded anaerobically. **b:** Full-range chromatograms from the anaerobic gel filtration of enzymatically reconstituted ISCU2 and its Y35 variants presented in Fig. 5d (green dotted boxes).

Supplementary figure 10

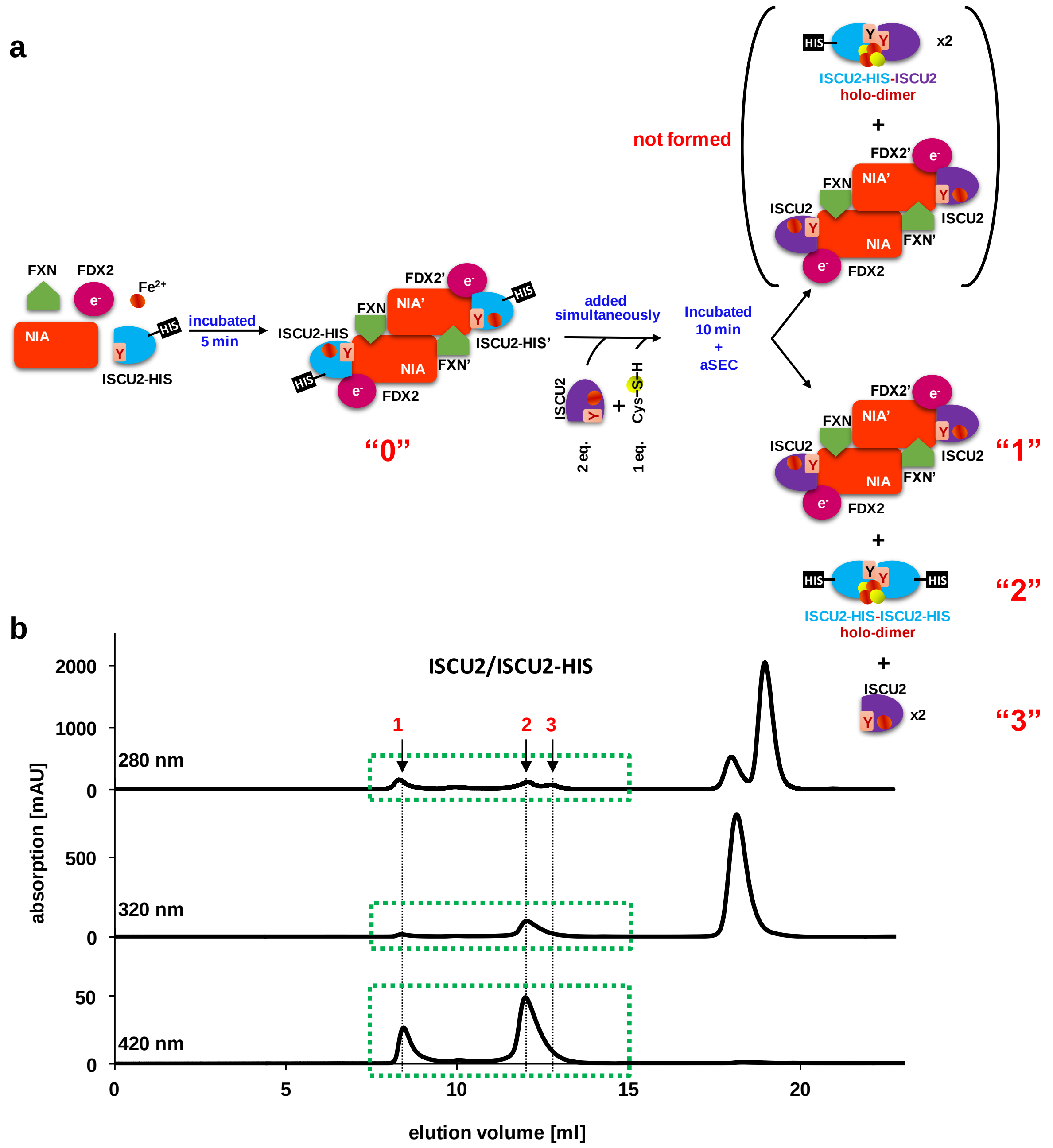

Supplementary Fig. 10: Free apo-ISCU2 does not contribute to holo-ISCU2 dimer formation. a: Experimental setup for Fig. 6b. $(NIA)_2$, ISCU2-HIS, FXN and FDX2 were incubated for 5 min (sample "0"), and subsequently free cysteine (1 molar equivalent to ISCU2-His) and non-tagged apo-ISCU2 (2 molar equivalents to ISCU2-His) were added simultaneously. After 10 min of incubation and subsequent analytical size exclusion chromatography (aSEC) samples "1", "2" and "3" were collected. **b:** Full chromatograms of aSEC shown in Fig. 6b (green dotted boxes).

Supplementary Tables

Supplementary Table 1: Quantification of Western blotting results from Fig. 1b. Signals were determined in relation to $F_1\alpha/\beta$ ATP synthase as a loading control, and expressed relative to values of mock-transfected cells (pSUPER/pEGFP-N3, set to 100 %) (mean ± SD; n≥3).

**depletion time**	**3 days**					**6 days**				
**pSUPER**	+	-	-	-	-	+	-	-	-	-
**pEGFP-N3**	+	+	-	-	-	+	+	-	-	-
**shISCU**	-	+	+	+	+	-	+	+	+	+
**smISCU2**	-	-	+	-	-	-	-	+	-	-
**smISCU2ΔMLS**	-	-	-	+	-	-	-	-	+	-
**smISCU2_Y35A**	-	-	-	-	+	-	-	-	-	+
**α-IRP1**	100	84±17	92±22	64±17	57±12	100	75±12	102±37	68±16	53±33
**α-GPAT**	100	29±17	97±29	35±11	51±17	100	13±6	119±46	19±12	37±19
**α-DPYD**	100	26±10	61±18	28±5	32±3	100	16±9	58±19	25±7	22±16
**α-SDH**	100	40±12	92±15	41±12	40±12	100	23±3	106±26	22±6	25±8
**α-mtAco**	100	70±14	99±34	79±51	98±47	100	45±24	129±28	49±5	48±19
**α-FeCh**	100	37±5	93±20	49±16	54±24	100	17±4	90±19	24±9	35±10

Supplementary Table 2: Data collection and refinement statistics of crystallographic experiments.

	NFS1-ISD11-ACP-ISCU2 M140I	NFS1-ISD11-ACP-ISCU2 WT	NFS1-ISD11-ACP-ISCU2 L35	NFS1-ISD11-ACP-ISCU2 L35H36	NFS1-ISD11-ACP-ISCU2 Y35D
Wavelength	0.97857	0.97857	0.97857	0.97857	0.97857
Resolution range	43.33-1.57 (1.63-1.57)	48.93-1.8 (1.86-1.8)	49.01-1.95 (2.02-1.95)	48.97-1.9 (1.97-1.9)	49-2.5 (2.59-2.5)
Space group	$P\ 4_1\ 2_1\ 2$	$P\ 4_1\ 2_1\ 2$	$P\ 4_1\ 2_1\ 2$	$P\ 4_1\ 2_1\ 2$	$P\ 4_1\ 2_1\ 2$
Unit cell (Å)	a = 86.41<br>b = 86.41<br>c = 245.79	a = 86.35<br>b = 86.35<br>c = 245.36	a = 86.43<br>b = 86.43<br>c = 246.12	a = 86.35<br>b = 86.35<br>c = 245.98	a = 86.35<br>b = 86.35<br>c = 246.37
Reflection measured	1,887,028 (186,230)	1,273,406 (119,988)	1,816,946 (178,339)	1,959,164 (196,690)	429,784 (41,359)
Unique reflections	130,412 (12,829)	87,545 (8566)	69,004 (6795)	74322 (7325)	33,256 (3257)
Multiplicity	14.5 (14.5)	14.5 (14.0)	26.3 (26.2)	26.4 (26.9)	12.9 (12.7)
Completeness (%)	99.98 (99.9)	99.94 (99.73)	99.97 (100)	99.98 (99.99)	99.95 (99.94)
Mean I/sigma (I)	19.59 (2.14)	12.19 (2.66)	22.62 (4.12)	17.66 (3.54)	14.77 (2.61)
Wilson B-factor	21.56	20.71	30.61	28.15	44.06
R-merge	0.088 (1.535)	0.158 (1.043)	0.107 (0.965)	0.138 (1.07)	0.15 (1.04)
CC 1/2	0.999 (0.725)	0.997 (0.835)	0.999 (0.922)	0.999 (0.909)	0.998 (0.806)
Reflections used for refinement	130,411 (12,829)	87,524 (8565)	68,997 (6795)	74,316 (7325)	33,248 (3257)
R-work	0.16 (0.25)	0.16 (0.24)	0.16 (0.20)	0.16 (0.22)	0.18 (0.27)
R-free	0.18 (0.27)	0.19 (0.26)	0.18 (0.24)	0.18 (0.24)	0.22 (0.3)
Non-hydrogen atoms	6265	6266	6135	6194	5799
Macromolecules	5381	5393	5348	5351	5231
Ligands	494	486	380	406	358
Protein residues	683	682	688	687	678
RMS (bonds)	0.02	0.02	0.02	0.019	0.005
RMS (angles)	1.84	1.74	1.89	1.84	0.65
Ramachandran plot Favored (%)	97.91	97.61	96.90	97.49	97.31
Allowed (%)	1.94	2.39	2.65	2.22	2.69
Outliers (%)	0.15	0	0.44	0.30	0
Rotamer outliers (%)	0.7	0.52	1.58	2.97	1.28
Clashscore	14.15	12.75	13.08	21.54	4.71
Average B-factor	31.99	30.07	41.96	37.43	49.25
Macromolecules	29.39	27.94	40.13	35.53	48.48
Ligands	56.31	50.90	65.46	57.99	63.61
No of TLS groups	4	4	4	4	4
PDB code	6UXE	6W1D	6WI2	6WIH	7RTK

Supplementary Table 3: Oligonucleotides used for *in vivo* studies in human cell culture.

Sequence(s)	Description
5'-CAGCAUGUGGUGACGUAAU-3'	shRNA for ISCU depletion
Fwd:5'-CAGCATGCGGAGATGTTATGAAATTACAGATTC-3'<br>Rev:5'-GAGGCAGAGAAGAAATGATGTACAGGC-3'	Generates silent mutations in the coding region of huISCU2 targeted by shISCU
Fwd:5'-GACTCGCGCACAAGAAGGTTGTTGATCATTATG-3'<br>Rev:5'-GGCCCCGGCCCGACTCGCGCACAA-3'	Exchanges the nucleotide triplet TAT encoding Tyr35 by the alanine-encoding triplet GCG ("smISCU2_Y35A"). Codon exchange is underlined.
Fwd:5'-TAGGTACCATGGCCTATCACAAGAAGGTTGTTG-3'<br>Rev:5'-GAGAGGCAGAGAAGAAATGATGTACAGGC-3'	Generate huISCU2 lacking mitochondrial localization sequence, add Kozak sequence, new Met and Ala residues in front of Y35, and KpnI and BsrGI restriction sites for cloning smISCU2 into pEGFP-N1 ("smISCU2ΔMLS").

Supplementary Table 4: List of human mitochondrial ISC proteins recombinantly expressed in and purified from *E. coli*. The proteins were used for *in vitro* experiments in this study. For variants of ISCU2 and NFS1 the vector backbone and expression system as well as the tag (only ISCU2) were kept as in wild-type proteins.

Protein name	UniProt ID	Sequence	Vector backbone (for *E. coli* expression)	Antibiotic
NFS1	Q9Y697-1	MSLRPLYMDVQATTPLDPRVLDAMLPYLINYYGNPHSRTHAYGWESEAAMERARQQVASLIGADPREIIFT<br>SGATESNNIAIKGVARFYRSRKKHLITTQTEHKCVLDSCRSLEAEGFQVTYLPVQKSGIIDLKELEAAIQPDT<br>SLVSVMTVNNEIGVKQPIAEIGRICSSRKVYFHTDAAQAVGKIPLDVNDMKIDLMSISGHKIYGPKGVGAIYIR<br>RRPRVRVEALQSGGGQERGMRSGTVPTPLVVGLGAACEVAQQEMEYDHKRISKLSERLIQNIMKSLPDV<br>VMNGDPKHHYPGCINLSFAYVEGESLLMALKDVALSSGSACTSASLEPSYVLRAIGTDEDLAHSSIRFGIG<br>RFTTEEEVDYTVEKCIQHVKRLREMSPLWEMVQDGIDLKSIKWTQH	pET-Duet (MCSI)	Amp
ISD11	Q9HD34	MGSSHHHHHHGSPTTENLYFQGHNMAASSRAQVLALYRAMLRESKRFSAYNYRTYAVRRIRDAFRENK<br>NVKDPVEIQTLVNKAKRDLGVIRRQVHIGQLYSTDKLIIENRDMPRT	pET-Duet (MCSII)	Amp
ACP1	O14561	MGSDMPPLTLEGIQDRVLYVLKLYDKIDPEKLSVNSHFMKDLGLDSLDQVEIIMAMEDEFGFEIPDIDAEKL<br>MCPQEIVDYIADKKDVYE	pRSF-Duet (MCSI)	Kan
ISCU2	Q9H1K1-1	MAYHKKVVDHYENPRNVGSLDKTSKNVGTGLVGAPACGDVMKLQIQVDEKGKIVDARFKTFGCGSAIASS<br>SLATEWVKGKTVEEALTIKNTDIAKELCLPPVKLHCSMLAEDAIKAALADYKLKQEPKKGEAEKKLEHHHHH<br>H	pET24b+	Amp
FXN	Q16595	(MHHHHHHSSGVDLGTENLYFQ)SNASGTLGHPGSLDETTYERLAEETLDSLAEFFEDLADKPYTFEDYDV<br>SFGSGVLTVKLGGDLGTYVINKQTPNKQIWLSSPSSGPKRYDWTGKNWVYSHDGVSLHELLAAELTKALK<br>TKLDLSSLAYSGKDA	pMCSG7	Amp
FDX2	Q6P4F2	MASDVVNVVFVDRSGQRIPVSGRVGDNVLHLAQRHGVDLEGACEASLACSTCHVYVSEDHLDLLPPPEE<br>REDDMLDMAPLLQENSRLGCQIVLTPELEGAEFTLPKITRNFYVDGHVPKPH	pET15b	Amp
FDXR	P22570	MGSSHHHHHHSQDPNSTQEKTPQICVVGSGPAGFYTAQHLLKHPQAHVDIYEKQPVPFGLVRFGVAPDH<br>PEVKNVINTFTQTAHSGRCAFWGNVEVGRDVTVPELREAYHAVVLSYGAEDHRALEIPGEELPGVCSARA<br>FVGWYNGLPENQELEPDLSCDTAVILGQGNVALDVARILLTPPEHLERTDITKAALGVLRQSRVKTVWLVG<br>RRGPLQVAFTIKELREMIQLPGARPILDPVDFLGLQDKIKEVPRPRKRLTELLLRTATEKPGPAEAARQASA<br>SRAWGLRFFRSPQQVLPSPDGRRAAGVRLAVTRLEGVDEATRAVPTGDMEDLPCGLVLSSIGYKSRPVD<br>PSVPFDSKLGVIPNVEGRVMDVPGLYCSGWVKRGPTGVIATTMTDSFLTGQMLLQDLKAGLLPSGPRPGY<br>AAIQALLSSRGVRPVSFSDWEKLDAEEVARGQGTGKPREKLVDPQEMLRLLGH	pET-Duet (MCSI)	Amp

Supplementary Table 5: List of primary antibodies used in this study.

Antibody	Source and validation	Dilution used
rabbit anti-ISCU1/2	Lill laboratory, validated in house by ISCU2 RNAi (10.1016/j.ajhg.2011.10.005 and this study)	1: 400
rabbit anti-NFU1	Lill laboratory, validated in house by NFU1 RNAi (10.1016/j.ajhg.2011.10.005)	1:1000
mouse anti-IRP1	clone 295B; validated and provided by R. Eisenstein (Wisconsin, USA), detects an IRE-binding antigen (personal observation)	1:3000
rabbit anti-GPAT	affinity purified, validated (10.1093/hmg/ddm163) and provided by H. Puccio (Illkirch, France)	1:5000
rabbit anti-DPYD	Santa Cruz Biotechnology, cat. # sc-50521 (H-300), validated in house using DPYD-overproducing cells (personal observation)	1:200
rabbit anti-TOMM20	Santa Cruz Biotechnology, cat. # sc-11415 (FL-145), validated by the manufacturer and in house by cell fractionation (e. g. this study)	1:5000
mouse anti-Complex II subunit 30 kDa Ip (SDHB)	clone 21A11AE7, MitoSciences, cat. # MS203, validated by the manufacturer and in house by cell fractionation (e. g. this study)	1:1000
rabbit anti-mitochondrial aconitase	validated and provided by L. Szweda (Oklahoma, USA), detects antigen of predicted size in an organellar cell fraction (personal observation)	1:1000
rabbit anti-ferrochelatase	provided and validated by T. and H. A. Dailey (Georgia, USA), as well as validated in house by FECH RNAi (personal observation)	1:2000
rabbit anti-MIA40	validated and provided by J. Herrmann (Kaiserslautern, Germany), detects antigen of predicted size within the mitochondrial intermembrane space (10.1093/hmg/ddy183 and this study)	1: 1500
mouse anti-PDI	clone 34/PDI, BD Biosciences, cat. # 610947, validated by the manufacturer and by Cramm-Behrens et al. (10.1111/j.1600-0854.2008.00829.x)	1:750
mouse anti-phospho- H2AX	clone 3F2, Thermo Scientific, cat. # MA1-2022, validated by the manufacturer and in house by cell fractionation (e. g. this study)	1:5000
mouse anti-beta-actin	clone C4, Santa Cruz Biotechnology, cat. #sc-47778, validated by the manufacturer and by Bräutigam et al. (10.1073/pnas.1110085108)	1:1000
mouse anti-alpha tubulin	clone DM1A, Sigma-Aldrich, cat. #T9026, validated by the manufacturer and in house by immunofluorescence (personal observation)	1:10000
rabbit anti-Complex V subunits ATP5F 1A/B	raised against bovine ATP5F1A/B, validated and provided by H. Schägger and I. Wittig (Frankfurt, Germany)	1:1500
mouse anti polyHis	clone HIS-1, Sigma-Aldrich, cat. #H1029 (batch 0000093768), validated by the manufacturer and in house by immunoblotting against HIS-tagged proteins (e. g. this study)	1:10000

Supplementary References

1. Corpet, F. Multiple sequence alignment with hierarchical clustering. *Nucleic Acids Res.* **16**, 10881-10890. (1988).
2. Tong, W.H. & Rouault, T. Distinct iron-sulfur cluster assembly complexes exist in the cytosol and mitochondria of human cells. *EMBO J* **19**, 5692-700 (2000).
3. Braymer, J.J., Freibert, S.A., Rakwalska-Bange, M. & Lill, R. Mechanistic concepts of iron-sulfur protein biogenesis in Biology. *Biochim Biophys Acta Mol Cell Res* **1868**, 118863 (2021).
