# Supplementary material for: N-terminal tyrosine of ISCU2 triggers [2Fe-2S] cluster synthesis by ISCU2 dimerization
Source: Nat Commun. 2021 Nov 25;12:6902. doi: 10.1038/s41467-021-27122-w (PMC8617193; doi:10.1038/s41467-021-27122-w)
Supplement: Supplementary file 3 — Reporting Summary [file 41467_2021_27122_MOESM3_ESM.pdf]

nature portfolio

Corresponding author(s): Roland Lill, Miroslaw Cygler

Last updated by author(s): Nov 1, 2021

Reporting Summary

Nature Portfolio wishes to improve the reproducibility of the work that we publish. This form provides structure for consistency and transparency in reporting. For further information on Nature Portfolio policies, see our Editorial Policies and the Editorial Policy Checklist.

Statistics

For all statistical analyses, confirm that the following items are present in the figure legend, table legend, main text, or Methods section.

n/a	Confirmed	
☐	☒	The exact sample size (*n*) for each experimental group/condition, given as a discrete number and unit of measurement
☐	☒	A statement on whether measurements were taken from distinct samples or whether the same sample was measured repeatedly
☒	☐	The statistical test(s) used AND whether they are one- or two-sided<br>*Only common tests should be described solely by name; describe more complex techniques in the Methods section.*
☒	☐	A description of all covariates tested
☒	☐	A description of any assumptions or corrections, such as tests of normality and adjustment for multiple comparisons
☐	☒	A full description of the statistical parameters including central tendency (e.g. means) or other basic estimates (e.g. regression coefficient) AND variation (e.g. standard deviation) or associated estimates of uncertainty (e.g. confidence intervals)
☒	☐	For null hypothesis testing, the test statistic (e.g. *F*, *t*, *r*) with confidence intervals, effect sizes, degrees of freedom and *P* value noted<br>*Give P values as exact values whenever suitable.*
☒	☐	For Bayesian analysis, information on the choice of priors and Markov chain Monte Carlo settings
☒	☐	For hierarchical and complex designs, identification of the appropriate level for tests and full reporting of outcomes
☒	☐	Estimates of effect sizes (e.g. Cohen's *d*, Pearson's *r*), indicating how they were calculated

Our web collection on statistics for biologists contains articles on many of the points above.

Software and code

Policy information about availability of computer code

Data collection	LRL-CAT 31-ID beamline at the Advanced Photon Source (Argonne National Laboratory, IL) and 08ID beamline at the Canadian Light Source, CMCF sector, Saskatoon, SK
Data analysis	Phaser MR within ccp4i package (v 7.1), COOT v 0.9.5, Phenix for refinement v 1.19, Origin 8G

For manuscripts utilizing custom algorithms or software that are central to the research but not yet described in published literature, software must be made available to editors and reviewers. We strongly encourage code deposition in a community repository (e.g. GitHub). See the Nature Portfolio guidelines for submitting code & software for further information.

Data

Policy information about availability of data

All manuscripts must include a data availability statement. This statement should provide the following information, where applicable:

- Accession codes, unique identifiers, or web links for publicly available datasets
- A description of any restrictions on data availability
- For clinical datasets or third party data, please ensure that the statement adheres to our policy

The crystallographic datasets generated and analyzed during the current study are available in the PDB data base, www.rcsb.org. PDB IDs are as follows: (NIAU2)2 with wild-type ISCU2: 6W1D [http://doi.org/10.2210/pdb6W1D/pdb]; (NIAU2)2 with ISCU2-M140I: 6UXE [http://doi.org/10.2210/pdb6UXE/pdb]; (NIAU2)2 with ISCU2-L35: 6WI2 [http://doi.org/10.2210/pdb6WI2/pdb]; (NIAU2)2 with ISCU2-L35H36: 6WIH [http://doi.org/10.2210/pdb6WIH/pdb]; (NIAU2)2 with ISCU2-Y35D: 7RTK [http://doi.org/10.2210/pdb7RTK/pdb]; (NIAU1)2: 5WKP [http://doi.org/10.2210/pdb5WKP/pdb]; (NIAU2)2 plus bound frataxin: 6NZU [http://doi.org/10.2210/pdb6NZU/pdb]. All other datasets generated and analyzed during the current study are either shown in the source data file or are available from the corresponding author on reasonable request.

Field-specific reporting

Please select the one below that is the best fit for your research. If you are not sure, read the appropriate sections before making your selection.

- [x] Life sciences
- [ ] Behavioural & social sciences
- [ ] Ecological, evolutionary & environmental sciences

For a reference copy of the document with all sections, see nature.com/documents/nr-reporting-summary-flat.pdf

Life sciences study design

All studies must disclose on these points even when the disclosure is negative.

Sample size	sample size (n) was counted and represents biological repetitions of the respective experiment
Data exclusions	No data were excluded from analyses.
Replication	All attemps at replication were successful. Number of replications is stated for each experiment individually.
Randomization	n/a because no clinical trials are included
Blinding	n/a because no clinical trials are included

Reporting for specific materials, systems and methods

We require information from authors about some types of materials, experimental systems and methods used in many studies. Here, indicate whether each material, system or method listed is relevant to your study. If you are not sure if a list item applies to your research, read the appropriate section before selecting a response.

Materials & experimental systems

n/a	Involved in the study	
[ ]	[x]	Antibodies
[ ]	[x]	Eukaryotic cell lines
[x]	[ ]	Palaeontology and archaeology
[x]	[ ]	Animals and other organisms
[x]	[ ]	Human research participants
[x]	[ ]	Clinical data
[x]	[ ]	Dual use research of concern

Methods

n/a	Involved in the study	
[x]	[ ]	ChIP-seq
[x]	[ ]	Flow cytometry
[x]	[ ]	MRI-based neuroimaging

Antibodies

Antibodies used

The following primary antibodies were used for immunoblotting:
rabbit anti-ISCU1/2:
Lill laboratory, validated in house by ISCU2 RNAi (10.1016/j.ajhg.2011.10.005 and this study), dilution 1: 400
rabbit anti-NFU1:
Lill laboratory, validated in house by NFU1 RNAi (10.1016/j.ajhg.2011.10.005), dilution 1: 1000
mouse anti-IRP1 :
clone 295B; validated and provided by R. Eisenstein (Wisconsin, USA), detects an IRE-binding antigen (personal observation), dilution 1: 3000
rabbit anti-GPAT:
affinity purified, validated (10.1093/hmg/ddm163) and provided by H. Puccio (Illkirch, France), dilution 1:5000
rabbit anti-DPYD:
Santa Cruz Biotechnology, cat. # sc-50521 (H-300), validated in house using DPYD-overproducing cells (personal observation), dilution 1: 200
rabbit anti-TOMM20:
Santa Cruz Biotechnology, cat. # sc-11415 (FL-145), validated by the manufacturer and in house by cell fractionation (e. g. this study), dilution 1: 5000
mouse anti-Complex II subunit 30 kDa Ip (SDHB):
clone 21A11AE7, MitoSciences, cat. # MS203, validated by the manufacturer and in house by cell fractionation (e. g. this study), dilution 1: 1000
rabbit anti-mitochondrial aconitase:
validated and provided by L. Szweda (Oklahoma, USA), detects antigen of predicted size in an organellar cell fraction (personal observation), dilution 1: 1000

rabbit anti-ferrochelatase:
provided and validated by T. and H. A. Dailey (Georgia, USA), as well as validated in house by FECH RNAi (personal observation), dilution 1: 2000
rabbit anti-MIA40:
validated and provided by J. Herrmann (Kaiserslautern, Germany), detects antigen of predicted size within the mitochondrial intermembrane space (10.1093/hmg/ddy183 and this study),
dilution 1: 1500
mouse anti-PDI:
clone 34/PDI, BD Biosciences, cat. # 610947, validated by the manufacturer and by Cramm-Behrens et al. (10.1111/j.1600-0854.2008.00829.x), dilution 1: 750
mouse anti-phospho-H2AX:
clone 3F2, Thermo Scientific, cat. # MA1-2022, validated by the manufacturer and in house by cell fractionation (e. g. this study), dilution 1: 5000
mouse anti-beta-actin:
clone C4, Santa Cruz Biotechnology, cat. #sc-47778, validated by the manufacturer and by Bräutigam et al. (10.1073/pnas.1110085108), dilution 1: 1000
mouse anti-alpha tubulin:
clone DM1A, Sigma-Aldrich, cat. #T9026, validated by the manufacturer and in house by immunofluorescence (personal observation), dilution 1: 10,000
rabbit anti-Complex V subunits ATP5F1A/B:
raised against bovine ATP5F1A/B, validated and provided by H. Schägger and I. Wittig (Frankfurt, Germany), dilution 1: 1500
mouse anti polyHis:
clone HIS-1, Sigma-Aldrich, cat. #H1029 (batch 0000093768), validated by the manufacturer and in house by immunoblotting against HIS-tagged proteins (e. g. this study), dilution 1: 10000
Peroxidase-conjugated goat, anti-rabbit (#170-6515) and anti-mouse (HRP #170-6516) antibodies (Biorad, Germany) were used as secondary reagents.

Validation: see above

Eukaryotic cell lines

Policy information about cell lines

Cell line source(s)	HeLa cells: European Collection of Authenticated Cell Cultures (ECACC), cat. #93021013
Authentication	Not authenticated
Mycoplasma contamination	Cell lines were not tested for mycoplasma contamination
Commonly misidentified lines (See ICLAC register)	n/a
