# Supplementary material for: N-terminal tyrosine of ISCU2 triggers [2Fe-2S] cluster synthesis by ISCU2 dimerization
Source: Nat Commun. 2021 Nov 25;12:6902. doi: 10.1038/s41467-021-27122-w (PMC8617193; doi:10.1038/s41467-021-27122-w)
Supplement: Supplementary file 4 — Source Data [file 41467_2021_27122_MOESM4_ESM.zip › freibert-et-al_uncropped-blots-gels.docx]

**Source data**

**N-terminal tyrosine of ISCU2 triggers**

**[2Fe-2S] cluster synthesis by ISCU2 dimerization**

Sven-A. Freibert, Michal T. Boniecki, *et al.*

**Uncropped western blots for Figure 1.** Areas framed by red dotted line were used in the respective figure

**Uncropped western blots for Supplementary Figure 2.** Areas framed by red dotted line were used in the respective figure

**Technical supplements for Figures 3-6 and Supplementary Figure 8**

**f**

**e**

**d**

**c**

**b**

**a**

**g**

**a:** Pictures of full Coomassie blue-stained gel from Fig. 3c. Red box indicates data shown. **b:** Growth of yeast Nfs1-W138A variant was taken form an agar plate containing additional data. For Fig. 3e only red boxes were used. **c:** Pictures of full Coomassie blue-stained gel from Fig. 4c. Red box indicates data shown. **d:** Pictures of full Coomassie blue-stained gels from Fig. 5b. Red boxes indicate data shown. **e:** Supplement for Fig. 5d. Monomeric ISCU2 was achieved by incubation of isolated dimeric ISCU2 (ai) with DTT overnight. Prior to size exclusion chromatography DTT-treated ISCU2 was supplemented with 4 equivalents of Fe^2+^ (FeCl_2_). **f:** Pictures of full western blot from Fig. 6a. Red box indicates data shown. **g:** Pictures of full Coomassie blue-stained gel from Supplementary Fig. 8c. Red box indicates data shown.
